# Protective effects of sodium ferulate on flap transplantation via anti-inflammatory modulation and oxidative stress inhibition

**DOI:** 10.1590/1414-431X2020e10520

**Published:** 2021-05-17

**Authors:** Y.D. Sun, Y.S. Gao, L.W. Xu, Y.F. Zhang, C. Cheng, K.C. Wei, Jian Lin, G. Chen, C.Y. Liu, Q.F. Li

**Affiliations:** 1 Department of Plastic Surgery, Shanghai Ninth People's Hospital, Shanghai Jiao Tong University School of Medicine, Shanghai, China; 2 Department of Plastic Surgery, Jiangsu Province Hospital of Traditional Chinese Medicine, Nanjing, China; 3 Department of Plastic Surgery, Changzheng Hospital of Navy Medical University, Shanghai, China; 4 Department of Orthopedics, Xinhua Hospital (Chongming) Affiliated to Medical College, Shanghai Jiao Tong University, Shanghai, China

**Keywords:** Sodium ferulate, Flap transplantation, Ischemia-reperfusion injury, Inflammation, Oxidative stress

## Abstract

Ischemia-reperfusion injury (IRI) has brought attention to flap failure in reconstructive surgery. To improve the prognosis of skin transplantation, we performed experimental IRI by surgical obstruction of blood flow and used sodium ferulate (SF) to prevent IRI in rats. After SF treatment, the morphological and histological changes of the skin flaps were observed by H&E and Masson's trichrome staining. We also detected the expression levels of COX-1, HO-1, and Ki67 by immunohistochemical and western blot analysis. Moreover, enzyme-linked immunosorbent assay was used to identify the content of tumor necrosis factor (TNF)-α, myeloperoxidase (MPO), malondialdehyde (MDA), and nitric oxide (NO) in peripheral blood and skin tissue. Compared with the model group, SF treatment significantly improved the recovered flap area (%) and promoted collagen synthesis. Cyclooxygenase-2 (COX-2) expression was significantly inhibited by heme oxygenase-1 (HO-1) induction after SF treatment. Furthermore, SF significantly inhibited the levels of TNF-α in peripheral blood, MPO and MDA in the skin tissue, and the increased synthesis of NO. Our results showed the protective effects of SF on IRI after flap transplantation and we believe that the protective effects of SF was closely related to the alleviation of the inflammatory response and the inhibition of the oxidative stress injury.

## Introduction

Flap transplantation is the main modality in wound repair and reconstructive surgery, and partial ischemia and necrosis of the flap is the most common complication in reconstructive surgery (1). Inadequate hemoperfusion and ischemia-reperfusion injury (IRI) after transplantation are considered to be the major cause of flap necrosis and failure (2,3). Therefore, an effective way to improve the recovery of skin transplantation is important (4,5). With the deepening of research on mechanisms of ischemia and hypoxia injury, IRI prevention and treatment are becoming the promising tools for us to explore.

Traditional Chinese Medicine (TCM) encompasses a wide range of practices and has gained excellent feedback from Asian people in their physiological restoration (6). Among them, angelica has been shown to improve blood circulation and eliminate stasis (7). Studies have shown that angelica has good therapeutic effects on hypoxia and ischemia injury (8 [Bibr B09]). Studies have proven that sodium ferulate (SF, the brand name is Angelicone) is one of the effective components of angelica extract, which is a type of new non-peptide endothelin receptor antagonist. SF has beneficial effects in microcirculation through activating the anti-inflammatory system, enhancing anticoagulation modulation, increasing the synthesis of nitric oxide (NO), and preventing lipid peroxidation injury (8–11).

SF has been widely used in the treatment of angina pectoris, cerebral infarction, and renal ischemic lesions. It was also proven to be effective in the treatment of diabetic foot and chronic skin ulcers (12,13). However, the effects of SF on flap transplantation remain unknown. In this study, we investigated the effects of SF on the percentage of the recovered flap area after IRI by establishing an animal model of skin flap transplantation in rats. We explored the related mechanism of SF effects and provided an experimental basis for its subsequent clinical application.

## Material and Methods

### Experimental animals

Male Sprague Dawley rats (6-8 weeks, 230-270 g) were purchased from the Experimental Animal Center of General Hospital of Nanjing Military Region and were subjected to experimental IRI. The animals were fed with mixed feed, given natural light exposure in a single cage with the temperature being controlled at about 25°C. The animals were adapted for one week before the experiment. At 8-10 weeks of age, the rats were randomly divided into 3 groups as follows: sham surgery (n=24), model group with experimental IRI (n=24), and SF treatment group (n=24). All animal procedures performed in this study were in accordance with the ethical standards of the Ethics Committee and ARRIVE guidelines. The approval number of Ethics committee is IACUC-2003125.

### Experimental reagents

SF was purchased from Nanjing Simcere (China, National Medicine Permit No. H20065557). Myeloperoxidase (MPO), NO, and malondialdehyde (MDA) detection kit were purchased from Nanjing Jiancheng Biotech Corp (China). The antibody of cyclooxygenase (COX-2), heme oxygenase (HO-1), and Ki67 were purchased from the British Abcam company. The ELISA test kit for tumor necrosis factor (TNF)-α in rats was purchased from Shanghai JiaTai Biotech Corp. (China).

### Experimental IRI in rats

Experimental IRI was induced by surgical intervention of the blood flow of the flap and the sham-operated rats were used as control. Briefly, we used 3% sodium pentobarbital for intraperitoneal anesthesia (0.15 mL/100 g body weight). In the sham group (CTL group), the flap was sutured *in situ* and no other treatment was performed. In the model and SF treatment groups, we used inferior epigastric artery skin flap as a flap ischemia-reperfusion injury model, with the area of the axial flap about 3×6 cm^2^. Thereafter, we blocked the blood flow with a microvascular clamp immediately after the establishment of the superficial epigastric flap for 8 h. After that, surgical microscopy was performed to confirm the recovery of blood supply. In the SF group, SF was administered into the abdominal cavity at 80 mg/kg per day on the day before surgery, and for 7 days after surgery (the concentration of SF solution was 1%, 2.5 mL). The rats in the model group were given the same amount of saline intraperitoneally according to the same procedure.

### Indicators

In each group, 6 rats were randomly selected for observation at 8, 24 h, and 3 and 7 days after IRI surgery. The percentage of the recovered flap area was calculated by the recovered area compared to the total skin flap area, and the total surface area of the flaps was traced. We analyzed the areas using the ImageJ software (NIH, USA). The flap tissue was equally divided along the central point of the flap (the diagonal intersection was the center), fixed with morphological method, and made into tissue homogenate to detect the levels of MPO, NO, and MDA by enzyme-linked immunosorbent assay (ELISA) and the nitro blue tetrazolium (NBT) method. At the same time, the peripheral blood of rats was collected, and ELISA was used to detect the level of TNF-α in serum.

### Histological and immunohistochemical assessments

After the experiment, the rats were killed under isoflurane anesthesia and the flap tissue was washed in PBS. Skin tissue was fixed with 4% paraformaldehyde (PFA) and embedded in paraffin. Serial longitudinal sections were cut with a thickness of 5 μm, then deparaffinized, rehydrated, and antigen retrieved. Nonspecific immunoreactivity was blocked by incubation with normal BSA serum for 1 h at room temperature, and sections were incubated with anti-COX2, anti-HO-1, and anti-Ki67 antibodies (1:1000), incubated overnight at 4°C, followed by treatment with the streptavidin-peroxidase kit according to the manufacturer's instructions (Sangon Biotech, China). The sections were also stained with hematoxylin and eosin (H&E) to evaluate the morphological changes of the flap. As a control, a subset of sections was incubated with PBS instead of the primary antibody. The expression of COX2, HO-1, and Ki67, represented by the staining intensity under ×400 magnification, was assessed by double-blind evaluation.

### Western blot analysis

Skin samples were homogenized in RIPA lysis buffer with 1% phosphatase and protease inhibitor cocktail (Sigma-Aldrich, USA). The mixture homogenates were centrifuged (12,000 *g*, 15 min, 4°C) and the supernatants were collected. Protein concentration was measured following Bradford's colorimetric method. Furthermore, the supernatants were mixed with a 5× SDS/PAGE sample buffer. Equal amounts of proteins (20 μg) were separated by a 10% SDS/PAGE gel and then transferred to PVDF membranes (Merck Millipore, USA). After blocking in a 5% BSA buffer for 1.5 h, membranes were incubated at 4°C overnight with specific primary antibodies for COX2, HO-1, and GAPDH at a dilution of 1:000 (Cell Signaling Technology, USA). After washing with TBST three times, membranes were incubated with a secondary antibody (Cell Signaling Technology). For visualization of the bands, all membranes were incubated with Immobilon Western Chemiluminescent HRP substrate (Millipore) for desired durations.

### Statistical analysis

All data were processed by SPPS (IMB, USA) 13.0 statistical software, and the data are reported as means±SD. Student's *t*-tests (unpaired, two-way) were applied to compare sham versus treatment groups. Statistical significance was set at P<0.05.

## Results

### SF treatment increased the recovered skin flap area

**Figure 1:**
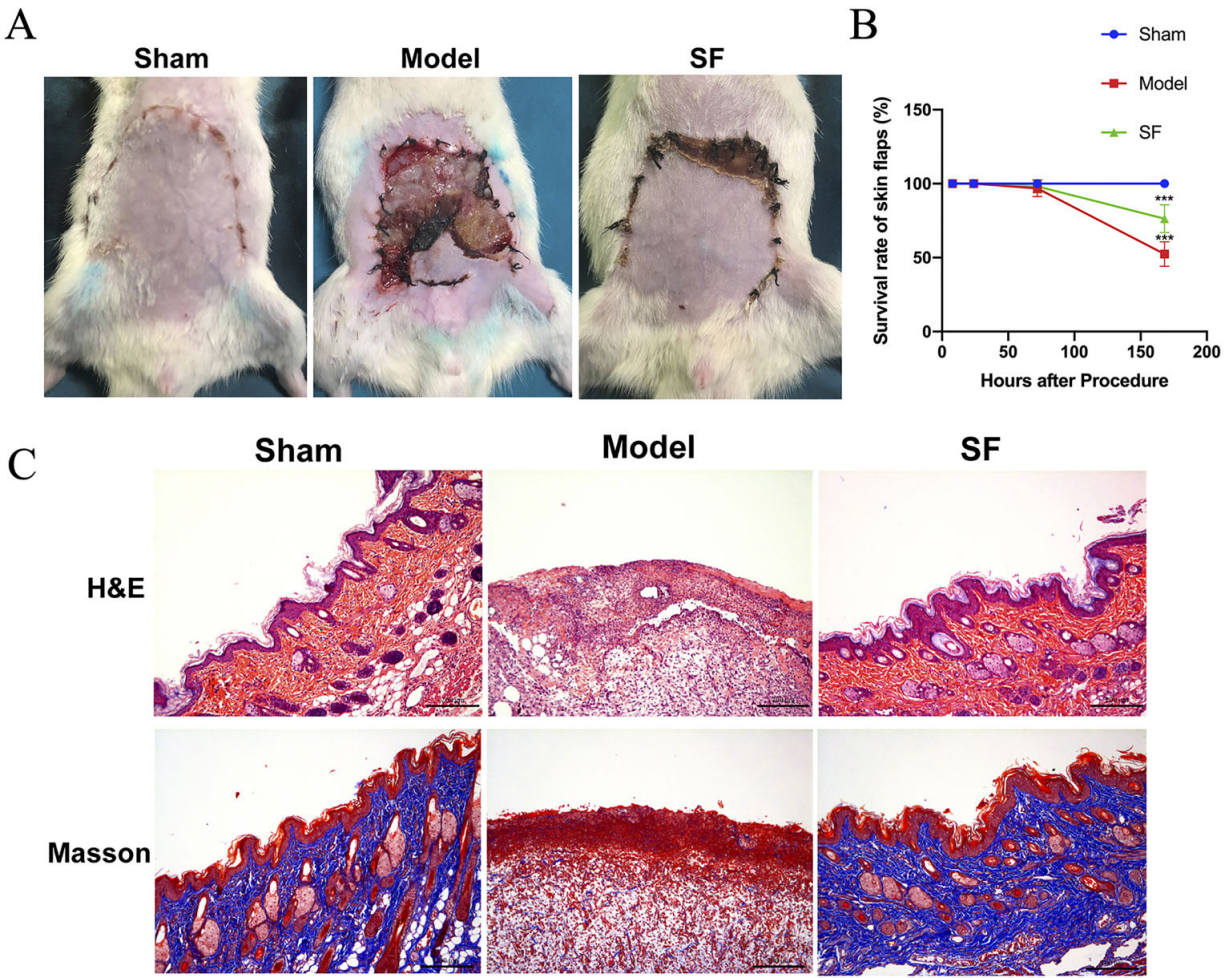
Morphological images of the **A**) lesion area and **B**) comparison of the percentage of the recovered skin flap area (%, mean±SD, n=6 per group) after 7 days of establishment of the ischemia-reperfusion model in each group (***P<0.001 compared to Sham, Student's *t*-test). **C**, H&E staining of the flap tissue (top). Masson staining (bottom) suggests the improved synthesis of the collagen after sodium ferulate (SF) treatment. Scale bar, 200 µm.

In the image from the control group, both the epidermis and dermis appeared normal and intact. Additionally, no animals died and no blood accumulation, effusion, or infection occurred in the sham group (Figure 1A). However, compared with the sham group, necrosis and limited recovered zones were observed in the distal end of the flap 3 days post-surgery in the IRI group. Seven days post-surgery, there was partial ulceration and scabs formed on the distal end of the flaps (Figure 1A). In contrast, after SF treatment, the necrosis area of the skin in the SF group was significantly less than that in the model group. The recovered flap area (%) also increased after SF treatment (Figure 1B). Therefore, SF treatment had a protective effect on preventing IRI, increasing the percentage of the recovered flap area (Figure 1).

### SF served as anti-inflammatory modulator

**Figure 2:**
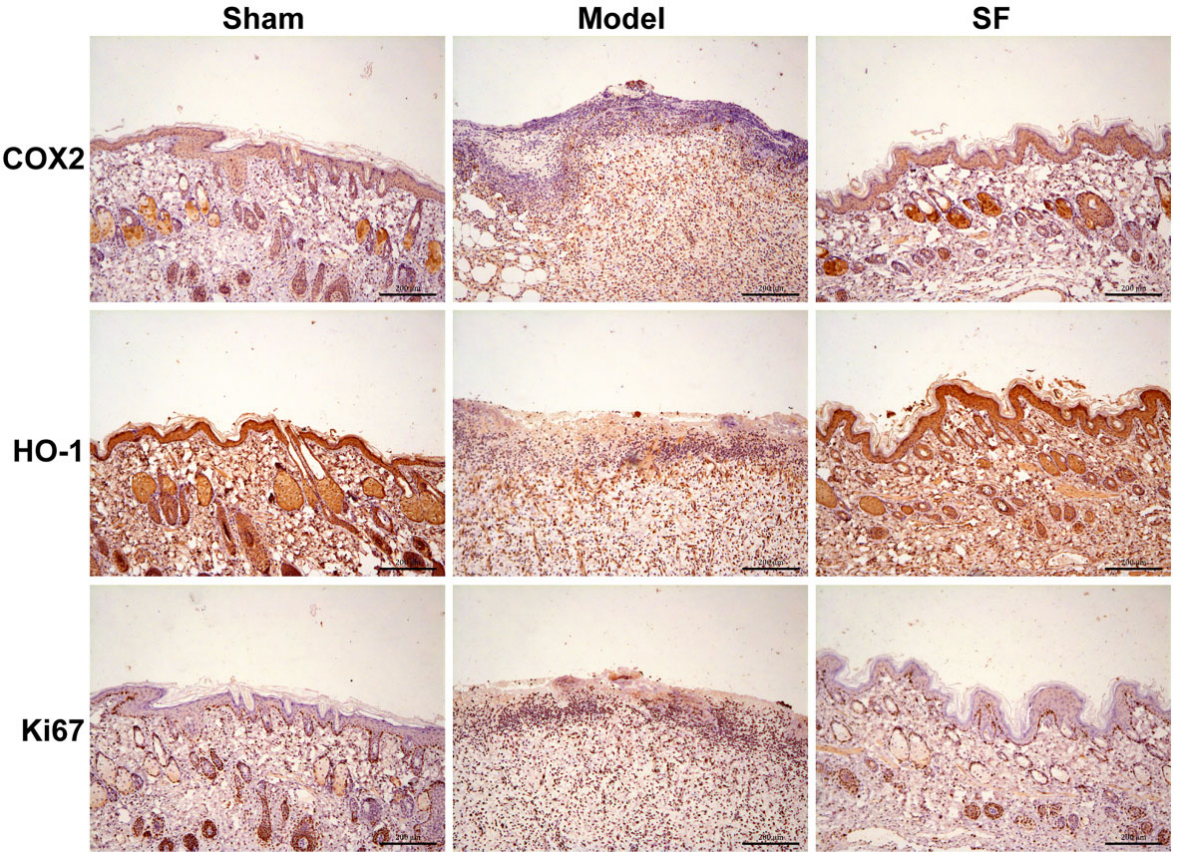
Assessment of the immunohistochemical staining of the flap tissue in each group: sham surgery, model group with experimental ischemia-reperfusion injury, and sodium ferulate (SF) treatment group. The SF group presented up-regulation of cyclooxygenase-2 (COX-2), heme oxygenase-1 (HO-1), and Ki67 markers compared with the model group. (n=6). Scale bar, 200 µm.

**Figure 3:**
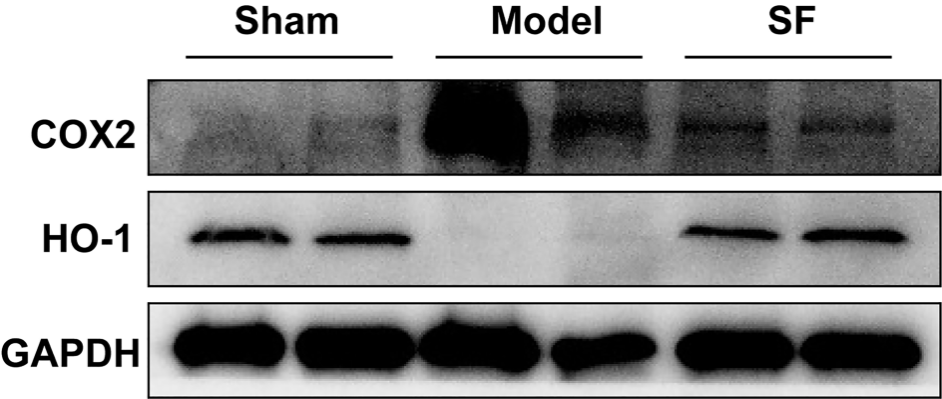
Western blot analysis results show the activation of cyclooxygenase-2 (COX2) expression and down-regulation of heme oxygenase-1 (HO-1) expression in model group (lanes 3 and 4) compared to Sham. After the sodium ferulate (SF) treatment, the results (lanes 5 and 6) were reversed and the COX2 and HO-1 expression levels were similar to the sham group (lanes 1 and 2).

In the model group, the inflammatory reaction of the skin graft was severe, with obvious inflammatory cell infiltration, and large necrotic areas compared with the sham group at different points of time after IRI (Figure 1C). However, at the same experimental time point, SF samples appeared histologically normal and the inflammatory reaction was attenuated. Masson's trichrome staining also revealed that SF prevented collagen loss and increased its synthesis after the IRI (Figure 1C). To confirm this finding, we tested two markers, COX-2 and HO-1, which play important roles in inflammatory reaction and oxidative stress response (14). Through COX-2 immunohistochemical staining, we observed that the degree of inflammation in the model group significantly increased after IRI surgery. In comparison, HO-1 decreased its expression due to severe inflammation (Figure 2). However, SF treatment resulted in a significantly decreased expression of COX-2 in the dermis, which means the inflammatory cytokines signal pathway was inhibited. Consistent with that, the expression level of antioxidant enzyme HO-1 was significantly increased in the SF group at 7 days after surgery. Consistent results were found by western blot analysis and SF showed outstanding protective effects to reverse the damage by IRI (Figure 3). These results suggested COX-2 expression was inhibited after SF treatment by HO-1 induction. Furthermore, SF had obvious regulatory functions on both inflammatory reaction and oxidative stress in the IRI model.

### IRI induced up-regulation of MPO, MDA, and NO and decreased TNF-&m_agr;

**Figure 4:**
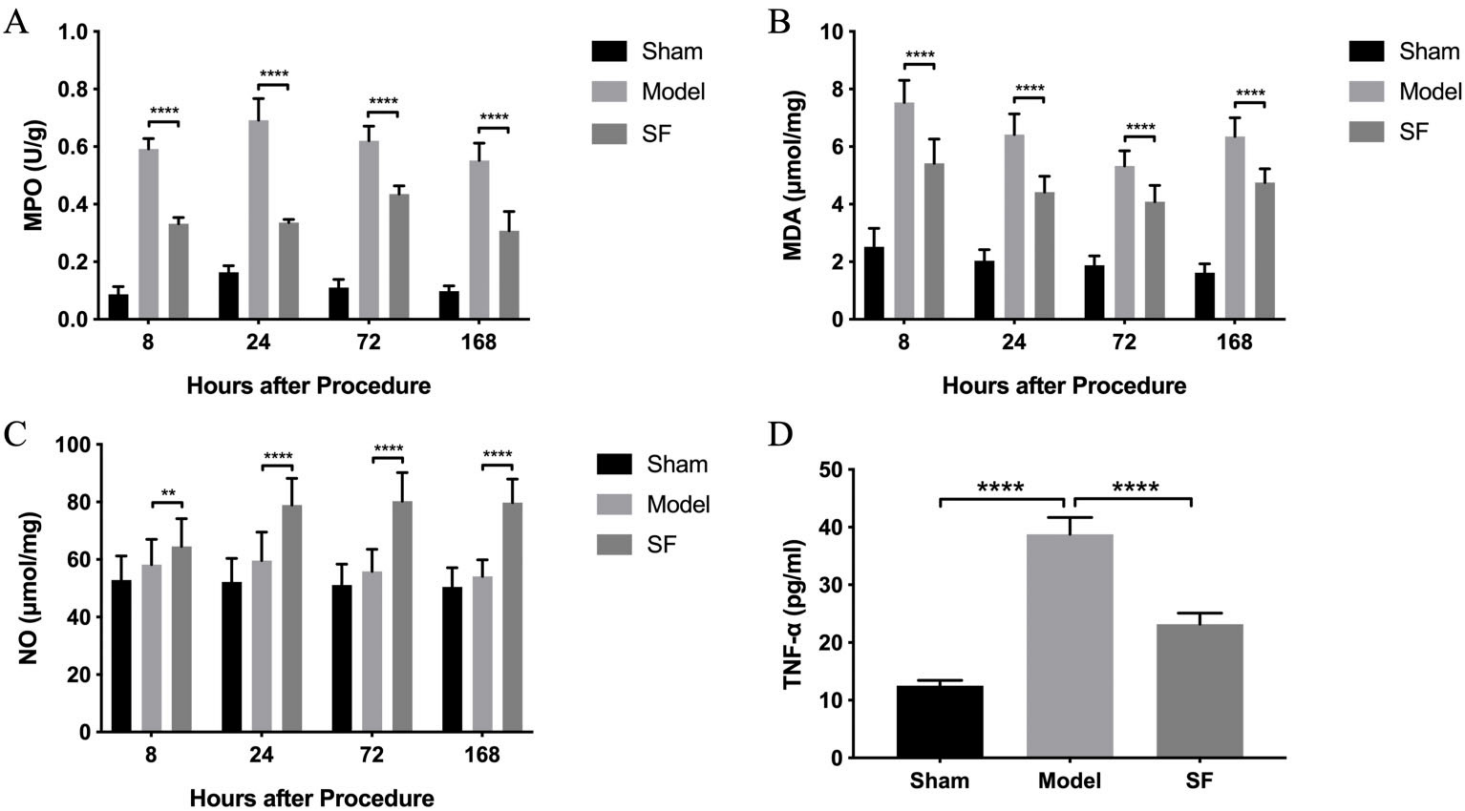
Myeloperoxidase (MPO), malondialdehyde (MDA), and nitric oxide (NO) changes in flap tissue. Tumor necrosis factor (TNF)-α levels in peripheral blood of sham surgery group, model group with experimental ischemia-reperfusion injury, and sodium ferulate (SF) treatment group (mean±SD, n=6). **P<0.05, ****P<0.001, Student's *t*-test.

We observed that the MPO and MDA concentrations increased after IRI surgery compared to the sham group (Figure 4A and B). Treatment with SF resulted in a significantly decreased expression of these two markers in tissue homogenate (P<0.05) (Figure 4A and B). In contrast, the NO concentration in tissues showed that the SF group was significantly higher than that of the model group and the sham group, while the difference between the model group and the sham group was not significant (Figure 4C). Additionally, at 24 h after surgery, the TNF-α concentration in peripheral blood was reduced significantly to a relatively normal level in the SF group (Figure 4D). These results further suggested that, consistent with our previous results, SF could efficiently protect against the inflammatory effects of IRI surgery shedding light on the positive effects of SF's application on IRI.

## Discussion

Regarding the molecular mechanism of flap IRI, research has shown that oxidative free radicals, intracellular calcium overload, microvascular injury, infiltration of inflammatory cells, and the release of related inflammatory cytokines play important roles in the ischemia-reperfusion process (15,16 [Bibr B17]). Oxygen free radicals can attack unsaturated fatty acids in biofilm and trigger lipid peroxidation to produce lipid peroxides, including aldehyde groups (MDA), keto, hydroxyl, carbon, and new free radicals, etc. When the release of free radicals exceeds the ability of the cell to repair itself, the cell can initiate the apoptotic pathway and ultimately cause tissue damage (710,).

SF is the sodium salt of ferulic acid (4-hydroxy-3-methylcinnamic acid). The formula of ferulic acid is C_10_H_10_O_4_ and its molecular weight is 194 (10). SF is a non-peptide endothelin receptor antagonist, which can inhibit endothelin-induced vasoconstriction, blood pressure and vascular smooth muscle cell proliferation, reduce vascular endothelial damage, inhibit platelet aggregation, scavenge free radicals, and prevent lipid peroxidation. In the cardiovascular and cerebrovascular systems, digestive system, urinary system, central and peripheral nervous systems, visual system, and in ischemia-reperfusion injury in both animal experiments and clinical studies, SF shows large potential and versatile protective roles (18,19).

This study confirmed that SF can significantly improve the recovered flap area (%) after IRI. Its mechanism may be due to the following aspects. First, SF has a role in the regulation of oxidative stress, which can improve the balance between NO and endothelin after IRI, and improve microcirculation (20). In normal tissues, endothelin and NO are in a state of balance. NO can counteract the vasoconstriction of endothelin, inhibit the formation of microthrombus, and avoid the occurrence of disseminated intravascular coagulation (8,). We found that the NO levels increased significantly in the SF group, and the mechanism of this phenomenon may be due to the specific endothelin receptor antagonist effect. Secondly, SF can reduce the formation of tissue MPO and MDA. MPO is the peroxidation product formed after polyunsaturated fatty acid metabolism, which is oxidized by the active oxygen *in vivo*. Therefore, the concentration of this marker can reflect the amount of free radicals produced *in vivo*. Thus, it also indicates the degree of damage of oxidative stress to the tissue. The results of this experiment showed that SF alleviated damage by oxygen free radicals and reduced lipid peroxidation injury caused by ischemia-reperfusion. In addition, SF reduced the inflammatory response caused by leukocyte infiltration. Leukocyte infiltration can mediate microvascular injury, which contributes to the pathogenesis of IRI. Neutrophils are activated, which adhere to vascular endothelial cells and cause the mechanical blockage of microvessels. They are also important sources of MPO, so an excess of neutrophils can increase MPO content in local tissues (8,).

This study found that during the process of IRI, the infiltration of inflammatory cells, such as neutrophils, in the flap tissue of the SF group was significantly decreased and the content of MPO was significantly decreased. In addition, the level of TNF-α in peripheral blood of the SF group was also significantly lower than the sham group, indicating that SF had a significant anti-inflammatory effect and inhibited neutrophil chemotaxis and aggregation in inflammation. Furthermore, SF may also inhibit cell apoptosis by reducing calcium overload. The protective effect of SF on a variety of cells has been reported. Liu et al. (21) has shown that SF relieves myocardial injury caused by Ca^2+^ overload, reduce myocardial calcium overload caused by IRI, and improve cardiac function. Li et al. (22) found that SF reduces the expression level of MDA and BAX protein in muscle tissue of the experimental group and reduces apoptosis after IRI. Although there is no direct evidence about the effect of SF on skin cells, in this experiment, we also observed that SF promoted the injured skin flap to recover histologically, indicating that SF may have a protective effect on normal skin cells.
